# Rat Cytomegalovirus Virion-Associated Proteins R131 and R129 Are Necessary for Infection of Macrophages and Dendritic Cells

**DOI:** 10.3390/pathogens9110963

**Published:** 2020-11-19

**Authors:** Iris K. A. Jones, Nicole N. Haese, Philippe Gatault, Zachary J. Streblow, Takeshi F. Andoh, Michael Denton, Cassilyn E. Streblow, Kiley Bonin, Craig N. Kreklywich, Jennifer M. Burg, Susan L. Orloff, Daniel N. Streblow

**Affiliations:** 1 Vaccine & Gene Therapy Institute, Oregon Health & Science University, Portland, OR 97239, USA; archeri@ohsu.edu (I.K.A.J.); haese@ohsu.edu (N.N.H.); strebloz@ohsu.edu (Z.J.S.); andot@ohsu.edu (T.F.A.); dentonm@ohsu.edu (M.D.); strebloc@ohsu.edu (C.E.S.); kileybonin@icloud.com (K.B.); kreklywi@ohsu.edu (C.N.K.); 2 Renal Transplant Unit, 10 Boulevard Tonnellé, University Hospital of Tours, 37032 Tours, France; philippe.gatault@univ-tours.fr; 3 Department of Surgery, Oregon Health & Science University, Portland, OR 97239, USA; jmburg.md@gmail.com (J.M.B.); orloffs@ohsu.edu (S.L.O.); 4 Department of Molecular Microbiology & Immunology, Oregon Health & Science University, Portland, OR 97239, USA

**Keywords:** cytomegalovirus, viral entry, dissemination, dendritic cells, chemokines, chemokine receptors

## Abstract

Cytomegalovirus (CMV) establishes persistent, latent infection in hosts, causing diseases in immunocompromised patients, transplant recipients, and neonates. CMV infection modifies the host chemokine axis by modulating chemokine and chemokine receptor expression and by encoding putative chemokine and chemokine receptor homologues. The viral proteins have roles in cellular signaling, migration, and transformation, as well as viral dissemination, tropism, latency and reactivation. Herein, we review the contribution of CMV-encoded chemokines and chemokine receptors to these processes, and further elucidate the viral tropism role of rat CMV (RCMV) R129 and R131. These homologues of the human CMV (HCMV)-encoded chemokines UL128 and UL130 are of particular interest because of their dual role as chemokines and members of the pentameric entry complex, which is required for entry into cell types that are essential for viral transmission and dissemination. The contributions of UL128 and UL130 to acceleration of solid organ transplant chronic rejection are poorly understood, and are in need of an effective in vivo model system to elucidate the phenomenon. We demonstrated similar molecular entry requirements for R129 and R131 in the rat cells, as observed for HCMV, and provided evidence that R129 and R131 are part of the viral entry complex required for entry into macrophages, dendritic cells, and bone marrow cells.

## 1. Introduction

Cytomegalovirus (CMV) is a β-herpes virus that establishes persistent latent infection in hosts, and causes severe diseases in immunocompromised patients. Transplant recipients, in particular, face impacts from CMV infection, if either the donor or the recipient are infected [1,2]. Although anti-viral prophylactic therapies, such as ganciclovir, are clinically used to control CMV in transplant recipients, these therapies are prone to generation of resistance mutants and do not protect against CMV-induced acceleration of chronic rejection (CR) and development of transplant vascular sclerosis (TVS) in latently infected grafts [2,3]. As such, a more thorough understanding of CMV dissemination and latency is necessary to guide the development of novel therapies and vaccine candidates. CMV dissemination within the host is a complex process involving regulation of host immune cells and cell-specific entry mechanisms. In order to regulate trafficking of the host immune cells to promote its dissemination, CMV uses virally encoded homologues of host chemokines and chemokine receptors. Hence, the focus of this research is to better elucidate the pathways involved in CMV viral entry into key immune cell populations that impact viral dissemination.

Chemokines (chemotactic cytokines) are a group of inducible cytokines that promote cellular migration and activation, by binding to their respective G-protein coupled receptors (GPCRs). The major chemokine groups are the CC chemokines, which include MCP-1, MIP-1α, MIP-1β, and RANTES; the CXC chemokines, which include IL-8, IP-10, and SDF-1α; the CX3C chemokines, which includes Fractalkine; and the C chemokines, which includes lymphotactin. Chemokines are generally involved in most aspects of immunity. Their binding to the receptors increases the cellular production of other cytokines and growth factors, and increases the expression of integrins promoting cellular adhesion to the vascular endothelia. Chemokines are present in the vascularized graft at all stages post-transplantation, including during ischemia/reperfusion injury, acute rejection, chronic rejection, and during the healing processes [4]. By contrast, long-term graft acceptance was attributed to the absence of chemokines, thus, substantiating a major role for chemokines in allogeneic graft rejection and during the development of TVS [5]. Both the CC and CXC chemokines were detected in human and experimentally induced animal models of graft rejection (reviewed in [4]). The CC-chemokines are produced as a result of vessel injury, which promote cellular adhesion to the endothelium, transmigration of immune cells, cellular activation, and migration. Chemokine receptors are present on all classes of immune and inflammatory cells and act as barcodes to direct immune responses. Chemokine ligand binding promotes signaling through G proteins and other signaling molecules to activate a diverse set of functional responses, including transcriptional activation and migration of the critical cells involved in inflammation and graft rejection.

All members of the β-herpesviruses encode chemokine and chemokine receptor homologues [6,7,8,9,10,11,12]. These modify host signaling and facilitate viral dissemination via their roles in entry and recruitment of cells to the site of infection, during CMV pathogenesis. A current list of the CMV-encoded chemokines and chemokine receptors are listed in Table 1 and Table 2, respectively. HCMV contains four putative GPCRs, which are encoded in the ORFs UL33, US27, US28, and UL78 [10]. RCMV and murine CMV (MCMV) only contain two putative chemokine receptor homologues R33 and R78, and M33 and M78, respectively. Interestingly, Rhesus CMV contains 7 chemokine receptors including 5 US28 homologues, a UL33 homologue, and a UL78 homologue. In addition, the β-herpesviruses HHV6 and HHV7 each encode UL12 (CC) and UL51 (CC) chemokine receptors. CMVs also encode at least four chemokine homologues, including UL128, UL130, UL146 (vCXCL-1), and UL147 (vCXCL-2).

### 1.1. Regulation of Host-Cell Signaling and Trafficking by CMV-Encoded Chemokine Receptors

The CMV encoded chemokine-receptor homologues are multi-functional, operating to promote viral infection through several unique mechanisms. CMV encoded chemokines and chemokine receptors display signaling functions that help to regulate cellular migration and immune-cell recruitment. Importantly, CMV encodes several chemokine and chemokine-receptor homologues that recruit or direct movement of macrophages and dendritic cells (DC), which might provide the virus with a vehicle for transmission and viral dissemination. Oral transmission of MCMV occurs through the infection of olfactory and alveolar epithelial cells. Infection of these cells then establishes infection of tissue-resident DC and macrophages [29,30]. Re-entry of these infected DC into the circulation is driven by the MCMV-encoded chemokine receptor M33. MCMV containing mutations in M33 fail to establish infection in the salivary glands, following intranasal infection, although the virus is capable of readily replicating at the initial site of infection [30,31]. Additionally, M33 promotes extravasation of infected DC into salivary gland tissues, explaining the loss of viral titer in salivary glands of M33-deficient mutants [32]. Importantly, replacement of M33 with the HCMV chemokine-receptor US28 also promotes infected DC to re-enter circulation from the site of infection [32]. This function appears to be highly conserved across the CMV species, as RCMV R33 mutants also fail to show viral replication in salivary glands [33]. However, in the case of R33, trafficking of virus did occur, but the virus failed to establish infection in the salivary gland tissue. R33-deficient RCMV also show reduced mortality in immunocompromised rats and delayed progression to chronic rejection in rat heart transplant recipients, compared to recipients infected with WT RCMV [33,34]. Importantly, these studies point to slight differences in the functionality of CMV-encoded chemokine receptors. However, US28 and UL33 are notably, partially redundant in function with MCMV M33, since they correct for a loss of MCMV reactivation and viral replication in salivary glands, in an M33-signaling deficient infection [35].

CMV chemokine receptors promote migration in other cell types as well. For example, US28 also promotes the migration of macrophages and vascular smooth muscle cells (vSMC) in a chemokine-dependent manner [36,37]. While US28 binds multiple chemokine ligands, signaling and migration are affected by ligand specificity [37]. Specifically, US28 induced migration of vSMC is driven by CC chemokine binding and is inhibited by Fractalkine. The opposite effect is observed in macrophages, wherein US28 migration is promoted by Fractalkine [37]. Coupling to Gα12/13 G proteins is critical for vSMC migration, as is signaling through Src and FAK [38,39]. Stable expression of US28 was also shown to increase migration of the HEK293 cells over the HEK293 cells expressing CX_3_CR1, in response to CX_3_CL1. Interestingly, this increase in migration is competitively inhibited by the CC chemokines CCL2 and CCL5, but not by CCL3 [40], which would support the binding of multiple chemokines by US28. M33 also drives migration of the infected cells, specifically mouse vSMCs, but not fibroblasts, in an mRANTES dependent manner [27]. Similarly, RCMV R33 is necessary for migration of infected vSMCs in the development of TVS, during chronic rejection of rat cardiac transplants [34]. Furthermore, US27 enhances CXCL12/CXCR4 signaling, suggesting that this protein might also have a role in monocyte recruitment and viral dissemination [41]. Interestingly, UL78 and homologues in other CMV species were not demonstrated to promote cellular migration, although R78 is expressed in macrophages and is required for efficient infection in the spleen [28].

In addition to its role in cellular migration, US28 was shown to be critical for HCMV latency and reactivation [42,43,44]. US28 ligand binding activity is critical for maintaining the virus in a latent state, but ligand specificity is still unknown. Interestingly, the vGPCR is also required to promote reactivation, which might be driven by the ability of US28 to promote myeloid lineage cellular differentiation [42]. US28 was also implicated in the suppression of IL-8 secretion and the sequestering of cellular/host chemokines, and exogenously expressed chemokines during CMV infection, thus regulating immune response to virally-infected cells [45]. UL33 was shown to facilitate cell–cell spread of HCMV, and loss of UL33 reduces viral titers in vitro in fibroblasts; however, the precise function of UL33 in this process is still unclear [46]. Additionally, UL78 is required for a step between virus binding and entry phases in epithelial cells. However, UL78 does not appear to be necessary for viral entry in fibroblasts [47]. Prior work also demonstrated that an RCMV virus expressing a null mutant form of R78 displayed lower replication efficiency in vitro and a lower lethality in vivo [28]. These studies suggest that CMV-encoded chemokine-receptor homologues function to increase viral dissemination via multiple potential mechanisms.

### 1.2. CMV-Encoded Chemokines Regulate Cellular Migration

HCMV UL128 and UL146 were shown to exhibit chemotactic activity. UL128 exhibits β-chemokine-like functions in its ability to recruit peripheral blood mononuclear cells (PBMC) [14]. In contrast, Straschewski et al. demonstrated that UL128 inhibits host-chemokine driven motility of monocytes and can cause monocyte paralysis [48]. This highlights the fact that even viral chemokines are responsive to cell-type specific differences. UL146 was shown to activate CXCR1, and, with a lower affinity, CXCR2, which might promote migration of neutrophils to the site of infection [18,19]. Furthermore, Heo et al. showed that there is a hyper-variability associated with UL146, which correlates with high functional selectivity in the recruitment and activation of neutrophils to infected tissues. UL146 induces Ca^2+^ flux and integrin expression on target cells, upon binding to host CXCR1 [49].

Studies in rats and mice demonstrated that CMV chemokine homologues contribute to immune cell migration to the site of infections, promoting further spread of the virus, in a manner similar to that seen with HCMV-encoded chemokines. Kaptein et al. showed that the putative UL130 homologue, R131, is involved in the recruitment of macrophages to the site of RCMV infection in rats [16]. Although lack of R131 does not seem to affect replication of RCMV in fibroblasts, null mutations in R131 correlate with a lack of a high titer of infection in the salivary glands of immunocompromised rats, and a significant decrease in footpad swelling, upon inoculation with RCMV [16]. It is worth noting that R131 has 41.1% sequence similarity with HCMV UL130 [17] and is predicted to be a CC-chemokine, rather than an XC-chemokine, and therefore, its chemokine functionality more closely resembles HCMV UL128. R129, the RCMV homologue of UL128, binds rat chemokine receptors CCR3, CCR4, CCR5, and CCR7 [13]. Additionally, migration of lymphocytes and naïve CD4^+^ T-cells were induced by r129 in in vitro transwell-migration assays [13]. RCMV containing an R129 mutation that lacks chemokine activity also failed to accelerate TVS and chronic rejection in a rat heart transplant model, indicating that the chemokine promotes CMV transplant disease through either its role as a chemokine or through participation in the pentamer receptor complex [13]. While deletion of the viral chemokines in the RhCMV strain 68.1 allows the virus to act as a potent viral vaccine vector [50], the role that these chemokines play in this process (chemotaxis vs. entry) is yet to be fully elucidated.

MCMV encodes a fusion product, MCK-2, from the MCMV genes m129 and m131, which are homologues of RCMV R129 and R131. MCK-2 also regulates the inflammatory response by inducing inflammation [51]. In a study by Fleming et al., Δm131/129 MCMV in vivo failed to produce high-titers in salivary glands and had improved clearance rates during acute MCMV infection from the spleen and liver in an NK cell- and T-cell-dependent manner. This finding suggests that m131/129 has pro-inflammatory properties and is necessary for immune evasion, by regulating NK and T-cells [52]. Further work in mice confirmed that MCK-2 enhances the recruitment of myeloid progenitors to the site of infection, which might aid in viral dissemination [53]. However, whether the effect of viral dissemination is limited to MCK-2′s ability to promote cellular migration or involves other mechanisms is yet to be determined. Additional in vivo studies suggested that MCK-2 mediates the recruitment of pro-inflammatory monocytes via CCR2, in order to impair CD8+ T-cell anti-viral responses, thereby slowing viral clearance [21]. Together these studies depict a clear relationship between the murine CMV-encoded 131/129 chemokine homologues and the promotion of pro-inflammatory conditions to promote viral dissemination. However, it was recently shown that MCMV self-regulates MCK-2 expression during infection via the virally-encoded M48 deubiquitinating enzyme, in order to regulate excessive inflammation associated with viral infection [54]. In a guinea pig model of CMV infection, deletion of gp1, a guinea pig CMV (GPCMV) homologue of the host chemokine MIP, allowed the generation of an immunogenic attenuated vaccine strain of GPCMV, which reduced viremia in non-pregnant guinea pigs and reduced DNAemia in the third trimester of pregnancy in guinea pig dams [55]. Intriguingly, work by Geyer et al. identified a novel XC chemokine (vXCL1) in the English strain of RCMV. vXCL1 recruits XCR1^+^ CD4^-^ dendritic cells in rats. Geyer et al. hypothesized that this allowed MuHV8 to undermine the traditional cytotoxic immune response [20]. In aggregate, regulation of leukocyte recruitment by CMV-encoded chemokines appears to promote viral dissemination and to inhibit viral clearance.

### 1.3. Role of CMV-Encoded Chemokines in Viral Entry

As mentioned above, CMV encoded chemokines play a role in viral entry through participation in the viral pentameric entry complex. Expression of different viral entry complexes determine cell tropism and can impact viral dissemination. HCMV encodes approximately 19 structural glycoproteins that are incorporated into the mature virion. However, not all of these glycoproteins participate in the viral entry process [56]. Of those that do, Glycoprotein B (gB), gH, gL, gM, gN, gO, UL128, UL130, and UL131A are the most well characterized for their roles in virion assembly and virus entry. These glycoproteins form several identified complexes including gB, gM/gN, gH/gL/gO (trimer), gH/UL116, and gH/gL/UL128/UL130/UL131A (pentamer) [57,58].

The gM/gN complex has roles in both viral entry and viral assembly, and the mutants are either non-viable or have severe replication deficiencies [56,59,60,61]. gB forms a functional homotrimer, which interacts with integrins and permits entry via pH-independent membrane fusion [62,63,64]. gH/gL forms the basis of two HCMV entry complexes, the trimer (gH/gL/gO) and pentamer (gH/gL/UL128/UL130/UL131A), which also function with gB to promote membrane fusion [65,66,67]. The trimer is essential for entry into fibroblasts, epithelial cells, and endothelial cells. The abundance of trimer incorporated into the virion correlates with infection levels in both fibroblasts and epithelial cells [68,69,70,71]. Trimer associated entry into fibroblasts involves binding of PDGFRα, followed by recruitment of gB [72,73,74]. The pentameric entry complex is unique among these glycoprotein complexes, in that it contains the viral chemokines UL128 and UL130, as well as UL131A. Pentamer-associated entry occurs in a pH-dependent manner [75]. The pentamer is not necessary for entry into fibroblasts, but is necessary for entry into epithelial cells, endothelial cells, dendritic cells, and monocytes [76,77,78,79,80]. Two receptors were recently identified for the pentamer—NRP2 in epithelial and endothelial cells and OR14I1 in epithelial cells [81,82].

While the pentameric entry complexes were studied for HCMV, little is known about the role of RCMV-encoded chemokines in viral entry. The RCMV 129 and 131 proteins are predicted to be putative homologues to the HCMV pentamer components UL128 and UL130, because they share chemotactic functions and positional homology with pentamer components from other CMV species [13,15,16]. In this report, we investigated the role that R129 and R131 play in viral entry and demonstrate that while the C’terminal domains (non-chemokine domain) are required for incorporation into RCMV particles, the R131 CC-domain is critical for mediating entry, suggesting a potential role in receptor binding of the R131 chemokine domain.

## 2. Results

### 2.1. C’terminal Truncations of R131 and R129 Fail to Incorporate into RCMV Particles

HCMV entry complex components are incorporated into the viral particle, in order to facilitate viral dissemination. In order to monitor viral protein incorporation of R129 and R131, we tagged each of these proteins with the 11 amino acid component (HiBiT) of the split NanoLuc protein-Lumit. The large portion could be added in trans solution- or membrane-based assays to reconstitute the enzyme and activate luminescence. We previously quantified the levels of R131 and R129 HiBiT incorporation into virus particles, and demonstrated that virion incorporated R131 and R129 are trypsin-sensitive, suggesting that both R131 and R129 are incorporated into the viral envelope [83]. However, the effect of further structural mutations and deletions on incorporation of R131 and R129 require additional study.

**Figure 1:**
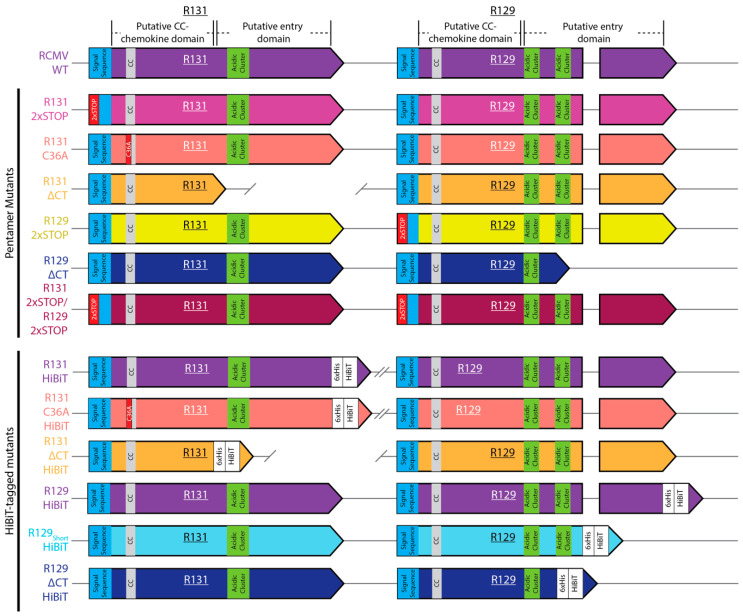
Construction of RCMV R131 and R129 mutants and HiBiT tag fusions. A panel of RCMV recombinants containing mutations in R131 or R129 was created using BAC recombineering. Mutants and HiBiT containing viruses are color-coded to the data graphs. As depicted, R131 consists of one exon, whereas R129 contains two exons and an intron. Putative domains of R131 and R129 are labeled on the WT version of both genes. Both R131 and R129 contain predicted signal sequences (blue), CC-chemokine domains (grey), and acidic clusters predicted to be involved in pentamer formation, based off of homology with the essential regions of HCMV UL130 and UL128, respectively (green). Truncation mutants were created by deletion of residues, and the 2xSTOP mutants and R131 C36A mutation are shown in red.

The HCMV homologues of R131 and R129, UL130 and UL128, have two unique domains, including an N’terminal chemokine-fold and a C’terminal region that interacts with other components of the pentamer entry complex. Charged clusters in UL128 and UL130 mediate incorporation of the proteins into viral particles, and their mutation alters entry phenotypes in human endothelial cells [84,85,86]. Structural data on the HCMV pentamer from Chandramouli et al. and phenotypic data on entry mutants from Schuessler et al. demonstrate that mutations in the α2 α-helix and β-sheets β4, 5, and 6 of UL128 are involved in interactions between UL128 and UL130. Mutation of the HSLTR sequence immediately preceding α2 or the EADGR sequence between β4 and β5 of UL128, result in severe entry impairments. Similarly, mutation of the UL128 KKHKR sequence following α3 and preceding Cys^162^, which interacts with gL, results in impaired entry into endothelial cells. In UL130, His^150^ in α4 allows for proper folding of UL130 and association with UL131A. Additionally, β4 and β5 interact with UL128 and UL131A and deletion of the DGTR sequence between the β-sheets and the HVFRD sequence partially contained in β5, result in severe entry impairments. His^209^ in the disordered C’terminal region of UL130 interacts with UL128, and Tyr^113^ in α2 of UL130 interacts with UL131A. There are charged residue clusters in both R131 and R129 that show homology to these charged regions of UL130 and UL128, respectively. To determine if loss of these regions altered incorporation of R131 and R129 into viral particles or viral tropism, truncation mutants were constructed by BAC recombineering, which excluded the acidic clusters and putative entry domains of the proteins, and added an in-frame HiBiT tag to the C’terminus of the proteins (Figure 1, ΔCT HiBiT). These truncations exclude the predicted homologous regions in R129 corresponding to the EADGR sequence, β5, β6, KKHKR sequence, and the disordered region containing Cys^162^ of UL128; or in R131, corresponding to α4 containing His^150^, β4, β5, the DGTR sequence, the HVFRD sequence, and the conserved His^209^ residue of UL130. To determine whether the loss of the CC-chemokine fold is necessary for virion incorporation or if it modulates cell tropism, we also constructed an RCMV mutant containing a HiBiT tagged R131, which contains an Alanine replacement of the first cysteine residue (C36) of the CC-motif (Figure 1, C36A HiBiT).

**Figure 2:**
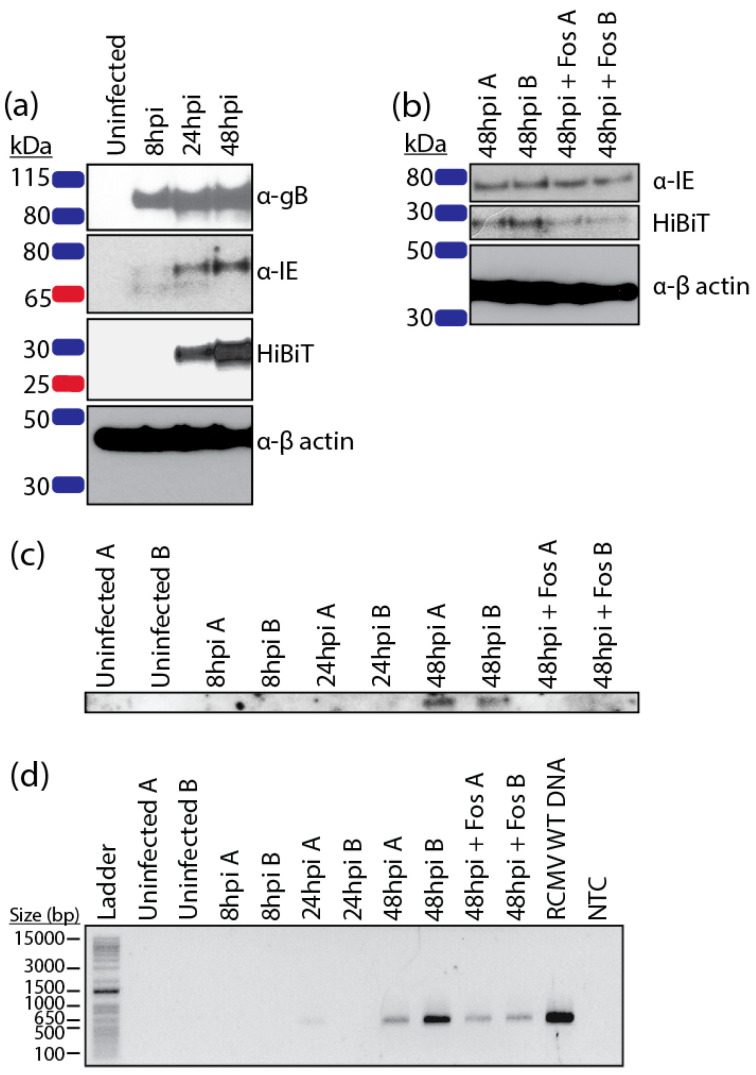
R131 is expressed with late viral gene expression kinetics. (**a**) Rat fibroblasts were infected with RCMV R131-HiBiT at a MOI = 1. Samples were washed with PBS and harvested in cell lysis buffer at 8, 24, and 48 hpi. Western blots for gB, RCMV IE, β-actin, and HiBiT (R131) were performed. (**b**) Rat fibroblasts were infected with RCMV R131-HiBiT at a MOI = 1 with or without foscarnet (0.5 mM) and samples were harvested in cell lysis buffer at 48 hpi. (**c**) Duplicate wells (A and B) of rat fibroblasts were infected with WT RCMV at an MOI = 1 with or without foscarnet (0.5 mM) and harvested in Trizol at 8, 24, and 48 hpi. RNA was isolated and Northern blots were performed probing for R131. (**d**) cDNA was made from RNA samples from (**c**) and reverse transcriptase PCR for R131 was performed. RCMV WT DNA was used as a positive control, water was used as the no template control (NTC). Size of select ladder bands are listed in base-pairs (bp).

HCMV, MCMV, and GPCMV express their pentameric entry complex proteins with late viral gene expression kinetics [23,87,88,89]. Previously, we demonstrated that RCMV R129 was also expressed with late viral expression kinetics and that expression was sensitive to foscarnet, an antiviral that targets the viral polymerase and prevents late gene expression [13]. In order to characterize R131 protein expression, we performed Western blots to detect the R131 HiBiT fusion protein in infected fibroblasts. R131 protein was detected by 24 hpi and accumulated up to 48 hpi (Figure 2a). Treatment with 0.5 mM foscarnet blocked R131 HiBiT expression at 48 hpi, suggesting that the protein is expressed with late viral gene expression kinetics (Figure 2b). Northern blots and rtPCR for R131 transcripts with RNA from infected RFL6 fibroblasts harvested at 8, 24, or 48 hpi also confirmed late viral gene expression at 48 hpi; and again, the expression was sensitive to treatment with foscarnet (Figure 2c,d). Northern blot analysis demonstrated that the viral gene is expressed as a single transcript at the predicted size of 700 nucleotides (Figure 2c).

**Figure 3:**
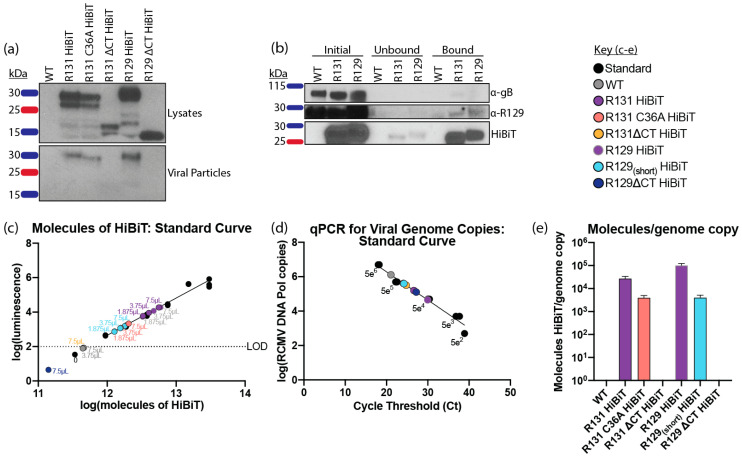
R131 and R129 C’terminal regions are required for viral incorporation. (**a**) Viral incorporation of R129 and R131 was assessed for wild-type RCMV and viral mutants containing R129 and R131 HiBiT tags. The viruses were grown in rat fibroblasts. At the time of maximum cytopathic effect, supernatants were harvested and cellular debris was removed by centrifugation. Viral particles were then pelleted by ultracentrifugation over a 10% sorbitol gradient, and the resuspended virus pellet was additionally purified by banding over a discontinuous histodenz gradient. The banded virus was collected by ultracentrifugation, over a 10% sorbitol gradient. Purified viral particles were resuspended in PBS and equivalent quantities of viral particles were determined by blotting for gB. Lysates of rat fibroblasts infected with RCMV HiBiT-tagged mutants were harvested in cell lysis buffer with protease inhibitors. Equal quantities of protein, as determined by the BCA assay, were loaded onto the SDS-Page gels and detected by the HiBiT blot with LgBiT. HiBiT tagged R129 and R131 was detected in infected cell lysates (upper panel) for all viruses, including mutants. While WT HiBiT tagged R129 and R131 as well as the R131 C36A mutant were present in viral particles, the C’terminal deletion mutants were excluded from the purified viruses indicating that they were not incorporated (lower panel). (**b**) Viral particles were prepared as described in (**a**) for WT RCMV and the R129 and R131 HiBiT-tagged viruses. The samples were normalized to an amount of gB using Western blot (initial sample). Equal quantities of gB-containing viral particles were subjected to pull downs utilizing LgBiT-Halo Tag protein bound to Halo-Tag magnetic beads. The unbound fractions and bead bound fractions were analyzed by Western blotting for gB, R129, and HiBiT. (**c–e**) C’terminal deletions of R131 and R129 are not incorporated into viral particles. (**c**) Three different volumes of each virus preparation (7.5 μL, 3.75 μL, 1.875 μL) were assayed in triplicate against a standard curve by HiBiT lytic detection assay. Molecules of HiBiT per μL of virus preparation was determined using a commercially available standard HiBiT-tagged protein. (**d**) Viral DNA was extracted from 12.5 μL of each virus preparation and DNA was diluted 1:1000 and analyzed in triplicates by qPCR, using primers and probes, directed against the RCMV DNA polymerase. A standard curve of known concentration RCMV DNA was used to determine viral genome copies in each sample. (**e**) Molecules of HiBiT over viral genome copies in each sample was compared. Data from (**c**,**d**) were normalized per μL of the initial virus preparations.

Next, we sought to confirm the expression of the HiBiT-tagged proteins R129 and R131 for the recombinant RCMV viruses containing mutations in the C’terminal domain and the CC motif. Rat fibroblasts were infected and lysates and supernatants were collected at the time of maximum cytopathic effect. Cell lysates and virus particles purified from the supernatants of the infected cells were analyzed by Western blotting for HiBiT. This analysis confirmed the presence of R129 and R131 HiBiT tagged viruses in the cell lysates and verified the deletion of the C’terminal domains (Figure 3a, upper panel). Interestingly, while the tagged proteins were detected for all viral mutants in the cell lysates, the C’terminal truncation mutants failed to be detected in the viral particle preparations (Figure 3a, lower panel). If R131 and R129 are members of the pentamer complex, we would expect that they should co-precipitate. Consequently, to determine whether R129 and R131 co-precipitate in samples of virus particles, we utilized a novel technique of HiBiT-based precipitation [90]. For this approach a Halo-tag reagent was used to couple LgBiT to magnetic beads that could be used to capture HiBiT-tagged R131. R129 was detected in the pull-downs using our previously generated polyclonal mouse antiserum that recognizes R129 [13]. Antibodies directed against gB were used to normalize the levels of WT, R131 HiBiT, and R129 HiBiT RCMV preparations. Equal quantities of gB-containing viral particles were lysed and incubated with LgBiT-HaloTag protein, and immunoprecipitation was performed using HaloTag beads. Using this method, both R131 and R129 could be pulled-down and detected using HiBiT (Figure 3b). Importantly, despite the lower dynamic range of detection seen with the α-R129 antibody, R129 was immunoprecipitated in both the R129 HiBiT control and the R131 HiBiT, demonstrating that R129 co-precipitated with R131. gB was not detected in the precipitated samples for WT, R131 HiBiT, or R129 HiBiT, and R129 did not bind to the HaloTag beads in the WT (negative control) samples. Since R131 and R129 were incorporated into viral particles and associated with each other, we next asked how many molecules of R131 and R129 mutants were incorporated into each virion, relative to the viral genomes. To address this subject, we performed the HiBiT lytic quantification assay on 3 different volumes of virus preparations (7.5 μL, 3.75 μL, and 1.875 μL) of each virus, in triplicate. We developed a standard curve of a known number of molecules of the HiBiT control protein available from Promega (Figure 3c). For each mutant, molecules of the HiBiT-tagged protein per microliter of virus preparation were determined. Genome copies per μL of each virus preparation were then determined by qPCR, using primers directed against the RCMV DNA polymerase gene (R54) (Figure 3d). Molecules of HiBiT-tagged protein per genome were calculated. R131 and R129 were incorporated at 2.6 × 10^4^ and 1.0 × 10^5^ copies per viral genome, respectively. Importantly, our quantification supported our earlier findings that the ΔCT mutants of R131 and R129 were not incorporated into the virion (Figure 3e) indicating that, similar to UL128 and UL130, the charged cluster rich C’terminal domain was necessary for incorporation into the pentamer complex [84,85]. Interestingly, both the R131 C36A and R129_(short)_ structural mutants were incorporated at slightly lower levels than the R131 and R129 WT proteins.

### 2.2. R131 and R129 are Required for Entry into Bone Marrow Cells, Dendritic Cells, and Macrophages

**Figure 4:**
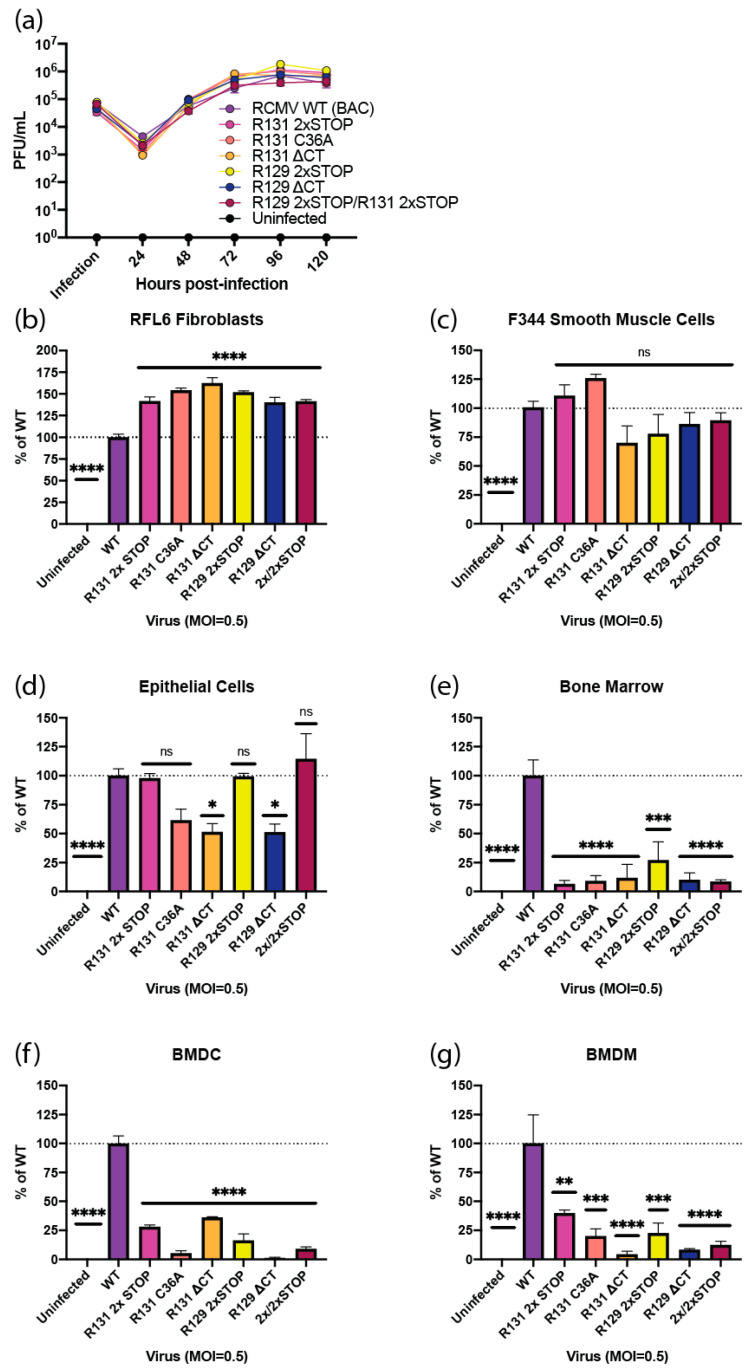
R131 and R129 are essential for entry into bone marrow, dendritic cells, and macrophages, but not fibroblasts or vascular smooth muscle cells. (**a**) Multistep growth curves were performed in triplicate wells by infecting rat fibroblasts with RCMV WT, R131 mutants, or R129 mutants at an MOI = 0.1. At 2 hpi, cells were washed three times with PBS and fresh medium was added to each well. Supernatant samples were collected at the time of infection and every 24 hpi until 120 hpi. The supernatants were titered by limiting dilution plaque assays in 24 well plates containing confluent monolayers of rat fibroblasts. The plates were fixed and stained after 7 days and viral titers were calculated. (**b**–**g**) For entry assays, 96 well plates containing rat fibroblasts (**b**), vascular smooth muscle cells (**c**), SMG-derived epithelial cells (**d**), bone marrow cells (**e**), bone-marrow derived dendritic cells (**f**) and bone-marrow derived macrophages (**g**) were infected with RCMV WT, R131 mutants, or R129 mutants at a MOI = 0.5 in triplicate wells. At 20 hpi, the cells were fixed and stained with an α-RCMV IE polyclonal antibody and counterstained with DAPI, in order to count cell nuclei. Percent infection was determined by counting the number of IE positive cells divided by the number of cell nuclei. Percent of infection relative to WT virus was determined for each cell type. Data are representative of two independent experiments, each performed in triplicates. Statistical significance compared to infection levels with WT RCMV was determined for each viral mutant by one-way ANOVA, using Dunnett’s correction for multiple comparisons. ns = not significant, * *p* < 0.05, ** *p* < 0.01, *** *p* < 0.001, **** *p* < 0.0001.

Other CMV pentamer complex mutants exhibit varying impacts on cellular entry and tropism, raising the question of which cell types require the RCMV pentamer complex for entry. A panel of RCMV viral mutants was generated using BAC recombineering, including an R131 2xSTOP, R131 C36A, R131ΔCT, R129 2xSTOP, R129ΔCT, and a double mutant R131 2xSTOP/R129 2xSTOP (Figure 1, Pentamer mutants). In order to determine the role of R131 and R129 in cellular entry, this panel of mutants was used. The R131 2xSTOP and R129 2xSTOP mutants allow for determination of the impact of complete loss of each of these proteins on cellular entry. Comparing these mutants to the R131 2xSTOP/R129 2xSTOP mutant, identifies redundancy in the functions of R129 and R131, with regards to viral entry. Finally, the R131ΔCT, R131 C36A, and R129ΔCT are expected to exhibit an inappropriate protein structure, which are predicted to impair protein–protein interactions. Notably, viral incorporation data indicate that the C36A R131 mutant protein is still incorporated into the viral particle, albeit at lower levels than WT, whereas R131ΔCT and R129ΔCT are not likewise incorporated (Figure 2a,e). Multistep growth curves were used to determine whether any of the viral mutations affected replication in rat fibroblasts, relative to the wild-type virus. All viral mutants demonstrated normal replication kinetics in fibroblasts, suggesting that no replication defect was generated by the R131 or R129 mutations (Figure 4a). CMV pentamer complexes dictate cell-type specific entry. In order to identify the cell types in which R129 and R131 are required for cellular entry, we performed an immunofluorescence-based assay that quantifies the percentage of cells expressing the viral immediate early protein, relative to the total cell number at 20 hpi. Wild-type RCMV and RCMV R131 and R129 viral mutants were used to infect rat fibroblasts, vSMC, epithelial cells, bone marrow, dendritic cells, and macrophages. R131 and R129 mutants show significantly increased entry into fibroblasts compared to WT, suggesting improved entry for these mutants (Figure 4b). WT RCMV infected 53.6% of fibroblasts in this assay. Similarly, entry into vSMC was not significantly impacted by mutations in either R131 or R129, with WT infecting 82.0% of all cells (Figure 4c). All R131 and R129 mutants were substantially lower in entry into bone marrow cells, dendritic cells, and macrophages, where WT infected 6.1%, 19.5%, and 34.9% of cells, respectively (Figure 4e–g). Additionally, the R131ΔCT and R129ΔCT mutants exhibited impaired entry into epithelial cells, and entry of the R131 C36A mutant was reduced, although not significantly (Figure 4d). In epithelial cells, WT infected 36.3% of cells. Since none of the 2xSTOP mutants exhibited impaired entry into epithelial cells, this suggests that R131 and R129 structural mutants disrupt cellular entry mediated by other entry complexes, by competing with gH during viral assembly. Correspondingly, a complete loss of the pentamer complex in deletion of R131 or R129 appears to be void of an impact in viral entry via this pathway. Further work is necessary to determine if competition for gH results in a significant impact on competition between viral entry complexes.

## 3. Discussion

In addition to modulating host chemokine and chemokine receptor expression, CMV also encodes many of its own viral associated factors. While viral chemokines and chemokine receptors are thought to have had their function and evolution derived from host gene capture events, their functions were modified and enhanced, in order to increase fitness and promulgation of the virus. These modifications included altered signaling patterns, enhanced chemokine binding breadth, and facilitated incorporation into cellular entry complexes. The overlapping roles of CMV-encoded chemokines and chemokine receptors in CMV cellular entry and virus transmission, further complicates the study of this sophisticated pathogen. This virus expresses chemokines and associated receptors that play roles in infection of epithelial cells and monocytes, leading to enhanced virus persistence and dissemination, as well as downstream damage to infected tissues and transplanted organs. Prior animal and in vitro studies with MCMV demonstrated that epithelial cells and monocytes are crucial for appropriate viral dissemination and subsequent downstream sequelae [29,30,31,91]. Given that the HCMV pentamer is required for entry into these cell types, further investigation of this complex in functional disease models is warranted.

The HCMV pentamer consists of the gH/gL scaffold, UL128, UL130, and UL131A [92]. The functions and components of the pentamer are not strictly conserved across CMV species, making it difficult to establish in vivo models of CMV cellular entry. Importantly, both Rhesus and Guinea pig CMV entry complexes seem to closely mirror those of HCMV [93,94,95]. However, MCMV shows less functional homology [51,96]. Variants of the gM/gN, pentameric, and trimeric complexes were identified in RhCMV, GPCMV, and MCMV [93,94,96,97,98,99,100,101,102]. The RhCMV pentamer consists of gH/gL/Rh157.5/Rh157.4/Rh157.6 and is required for entry into epithelial cells, but not fibroblasts [93,103,104]. Similarly, the GPCMV pentamer consists of gH/gL/GP129/GP131/GP133 and is essential for entry into monocytes and endothelial cells, but not fibroblasts [94,95,105,106,107]. Additionally, GPCMV pentamer mutants show impaired entry into epithelial cells [105]. The predicted homologous complex in MCMV contains three known members, gH, gL, and MCK-2, where MCK-2 is a fusion product of the m129 and m131 genes [96]. The MCMV gH/gL/MCK-2 complex is not required for entry into fibroblasts, but is required for entry into macrophages [96,108]. In contrast to the HCMV pentamer, gH/gL/MCK-2 is not required for entry into epithelial cells, and mutants show an increased capacity to infect epithelial cells [96]. Although RCMV homologues of gH, gL, gB, gO, and gM were identified [15], the pentamer components remain to be experimentally determined. The data we present supports the predicted role of R131 and R129 in formation of a functionally homologous pentamer cellular entry complex, which results in significant impacts on infected hosts, with respect to RCMV’s pathogenesis and other associated pathological effects.

Our studies showed that R131 and R129 were both incorporated into viral particles at near equivalent molecular levels. Importantly, charged cluster domains within UL130 and UL128 are involved in appropriate formation of the HCMV pentamer, and mutation of these clusters results in impaired entry into endothelial cells [84,85]. Prediction of similar charged clusters in R131 and R129 resulted in recognition of acidic clusters, following the predicted chemokine N-loop domains of the proteins (Figure 1). Deletion of the acidic cluster regions present in the C’terminal domains of R129 and R131, resulted in a failure of the proteins to be incorporated into viral particles. In contrast, partial removal of the C’terminal region of an R129 mutant that retains the two acidic clusters (R129_(short)_) showed only a minor decrease in viral incorporation. This data demonstrated that the C’terminal region is required for virion incorporation. Interestingly, a mutant of the CC-motif of R131 (C36A) was incorporated into virions but failed to enter macrophages and dendritic cells, indicating that a functional R131 is required for entry. This might indicate that either gross structural changes of either R131 or the complex as a whole exist for this mutant or that the chemokine domain of R131 is necessary for entry receptor binding. NMR and cryo-EM studies aimed at determining structural changes resulting from these mutations would provide insight into the role of the chemokine fold in pentamer complex formation and function.

In order to determine if R131 and R129 associate in a complex, we performed HiBiT/HaloTag Co-precipitation experiments from purified viral particles. R129 successfully precipitated with R131, supporting the formation of a complex containing R131 and R129. Importantly, gB did not associate with either R129 or R131 in pull-downs. Although R131 and R129 were incorporated into the virion, a role for both proteins in entry remained to be demonstrated. The panel of R131 and R129 mutants exhibited normal growth kinetics in fibroblasts, as seen with other CMV pentamer mutants. R131 and R129 mutants also exhibited slightly above normal entry in fibroblasts and smooth muscle cells. Entry was significantly reduced for all R131 and R129 mutants in dendritic cells, macrophages, and bone marrow cells, which is consistent with pentamer mutants in other CMV species (Table 3). Previous studies identified CD34+ progenitor cells in bone marrow as a site of CMV latency [109,110]. Further work is necessary to determine if pentamer mutants show impaired abilities to establish latency. Of particular interest are the inconsistencies in epithelial cell entry requirements across CMV species. Although HCMV and RhCMV require the pentamer for epithelial cell entry, GPCMV and RCMV exhibit partial impairment to entry, while MCMV shows enhanced entry into epithelial cells, following mutation of MCK-2 [51,93,94,95,96,103,104,105,106,107,108]. Our data highlight an interesting difference in complete loss mutants of R131 and R129 and misfolded or C’terminal deletion mutants, where the complete loss mutants enter epithelial cells at similar levels to WT but the C’terminal mutants show impaired entry. These findings suggest that the potential for multiple entry mechanisms in epithelial cells might be impaired by competition for gH/gL scaffolds, in the case of structural mutants of R131 and R129. Such multiple mechanisms for epithelial cell entry might explain the differences seen in epithelial cell entry across different pentamer mutations and CMV species. Importantly, whether a fifth member of the RCMV pentamer exists, remains to be determined. Positional homology with the GPCMV genome would suggest R133 as a putative fifth member; however, this remains to be confirmed for RCMV.

CMV-encoded chemokines and chemokine receptors mediate multiple functions that are important for viral transmission and pathogenesis. Here, we demonstrated similar molecular entry requirements for R131 and R129 in rat cells as observed for HCMV, supporting the use of the RCMV rat cardiac transplant model, to study solid organ transplant rejection. Our data demonstrated a role for R131 and R129 as part of the viral entry complex required for entry into macrophages, dendritic cells, and bone marrow cells, depicting the evolution of viral chemokines to facilitate viral dissemination. These data advance comparisons between pentamer viral entry complexes amongst the common CMV model systems (Table 3).

## 4. Materials and Methods

Generation and Maintenance of cell cultures: The RFL6 rat fibroblast cell line (ATCC, CCL-192) were maintained in Dulbecco’s Modified Eagle’s medium (DMEM; ThermoFisher, Waltham, MA, USA), supplemented with 5% FBS and 100 U Penicillin/100 μg Streptomycin/292 μg/mL Glutamine (Fisher), at 37 °C in 5% CO_2_.

Generation of SMG-derived epithelial cells: Epithelial cells were isolated from F344 rat submandibular glands (SMG), using a protocol adapted from Beucler & Miller, 2019 [111]. In brief, rat submandibular glands were minced and digested in Dispase & Collagenase III (Sigma, St. Louis, MO, USA), at 37 °C for 3 h. The resulting cell suspension was filtered through a 70 μm filter and the cells were centrifuged at 216× *g* for 5 min. Red blood cells were lysed and the cells were washed in PBS. Cells were cultured in epithelial cell growth media (Cell Biologics, https://cellbiologics.com/) on a basement membrane matrix in 6-well plates. Salisphere growth was monitored, and after 5 days, the basement membrane matrix was digested with dispase/collagenase III solution. The cells were dissociated with trypsin and single cells were plated in epithelial cell growth media on tissue-culture-treated plates, for viral entry assays.

Generation of F344 bone marrow derived macrophages: Macrophages were differentiated from bone marrow collected from the femurs of naïve F344 rats. Bone marrow was collected by flushing bones with RPMI media (ThermoFisher, Waltham, MA, USA). The resulting cell suspension was filtered through a 70 μm filter and the cells were pelleted at 1500 rpm for 10 min. Red blood cells were lysed and the cells were washed once in RPMI. Cells were plated in 10% FBS RPMI with 25 ng/mL M-CSF (R&D Systems, Minneapolis, MN, USA) at 1 × 10^6^ cells/mL. Cells were allowed to differentiate for 7 days, before being scraped from plates and plated for viral entry assays.

Generation of F344 bone marrow derived dendritic cells: Dendritic cells were differentiated from bone marrow, as reported previously [112]. In brief, filtered bone marrow cells were plated in 10% FBS RPMI with 5 ng/mL IL4 (R&D Systems, Minneapolis, MN, USA) and 10 ng/mL GMCSF (R&D Systems, Minneapolis, MN, USA) at 1 × 10^6^ cells/mL. Cells were allowed to differentiate for 7 days, before being scraped from plates and plated for viral entry assays.

Generation of F344 vascular smooth muscle cells: vSMC cells were isolated from F344 rat aorta, as previously described [34]. In brief, a F344 rat aorta was minced in DMEM containing 10% FBS plus PSG (DMEM-10) and plated in 6-well dishes. The vSMCs vacated the tissue pieces and adhered to the tissue culture dish. Cells were expanded in DMEM-10 culture medium. Cells were stained with an α-SMC actin antibody to verify purity of the culture.

RCMV Bacterial Artificial Chromosome: The RCMV Maastricht strain genome was captured as a Bacterial Artificial Chromosome (BAC) containing enhanced green fluorescent protein (eGFP), using homologous recombination, by replacing ORFs r144–r146 with a BAC cassette [13,83]. A two-step recombination protocol was used to create all viral mutants. The 2xSTOP mutants were created by insertion of 2 STOP codons into the N’terminus of the appropriate open reading frame. Viruses containing in-frame fusions with the HiBiT tag (small component of the split nanoluciferase complex [90]) were constructed by insertion of the 11 amino acid tag at the C’terminus of the protein, or as indicated in Figure 1. Following the rescue and expansion of RCMV, virus preparations were aliquoted and stored at −80 °C. Viral manipulations were confirmed by sequencing and HiBiT-tag expression was verified by Western blotting. Viruses were titered by limiting dilution plaque assay, as described below.

Isolation of purified viral particles: RCMV viruses were expanded on RFL6 fibroblasts. At the time of maximum cytopathic effect, supernatants were harvested and clarified by ultracentrifugation (46,676.5× *g*), followed by filtration through a 70 μm filter. Virus was then pelleted over a 10% sorbitol cushion, resuspended in TNE buffer (50 mM Tris (pH 7.4), 100 mM NaCl, 10 mM EDTA), and banded via density gradient ultracentrifugation over a discontinuous 10–50% Histodenz gradient, and the banded virus was removed from the gradient. The virus fraction was then resuspended in PBS and pelleted over a 10% Sorbitol cushion. The pelleted virus was resuspended in a minimal volume of PBS, aliquoted, and stored at −80 °C, until use.

Plaque Assays: Viral supernatants and stocks were quantified by making serial dilutions ranging from 10^−1^ to 10^−6^. Confluent monolayers of the RFL6 cells in 24-well plates were incubated with the viral dilutions, for 2 h on a rocker, at 37 °C. After 2 h post infection (hpi), 250 μL of carboxymethyl cellulose diluted in culture medium was added to each well and cells were placed in a 37 °C incubator. At 7 days post infection (dpi) the cells were fixed with 3.7% formalin in phosphate buffered saline (PBS; ThermoFisher, Waltham, MA, USA) and stained with methylene blue. The viral plaques were counted to determine the viral titers.

RCMV multi-step viral growth curve: RCMV growth was assessed by multistep growth analysis in fibroblasts. Cells were plated at 1.5 × 10^5^ cells/well in 6-well plates and allowed to adhere overnight. Cells were infected at a multiplicity of infection (MOI), equal to 0.1 with RCMV WT, RCMV R131, and R129 mutants, or left uninfected as a control. At 2 hpi, the cells were washed 3 times with PBS to remove the unbound virus, and fresh DMEM-10 was added to the cells. At 24-h intervals beginning with 0 hpi, 100 μL samples of supernatant were taken. Plaque assays were performed on supernatants to quantify virus growth over the time-course. Infections were performed in biological triplicates. Statistical differences were determined by two-way ANOVA, with Tukey’s correction for multiple comparisons.

Antibodies: Rabbit anti-RCMV-IE polyclonal antibody, mouse anti-R129 polyclonal antibodies, and rat anti-RCMV gB monoclonal antibody were previously described [13,113,114]. All primary antibodies were used at a dilution of 1:1000 overnight at 4 °C. HRP-conjugated secondary antibodies (TrueBlot Rabbit α-Rabbit; TrueBlot Mouse α-Mouse; Southern Biotech α-Rat) were used at 1:10,000 dilution overnight at 4 °C, with blocking in 5% BSA-TBST. An HRP-conjugated anti-β actin antibody was used at a dilution of 1:10,000 with blocking in 5% BSA-TBST, as a loading control for Western blots.

Protein detection by western blotting: RFL6 cells were plated in 6-well dishes at 5 × 10^5^ cells/well and infected with RCMV at an MOI = 1 or left uninfected as a control. At 48 hpi, cell lysates were harvested in cell lysate buffer (Cell Signaling Technologies, Danvers, MA, USA) with 1× HALT (ThermoFisher Scientific, Waltham, MA, USA) and clarified by centrifugation at 9167× *g* for 10 min at 4 °C. Virus particles were prepared as described above. Cell lysates and viral particles were combined 1:1, with NuPage SDS running buffer (ThermoFisher, Waltham, MA, USA) + 2% β-mercaptoethanol and boiled for 7 min, then, centrifuged briefly to pellet debris. Proteins were separated on SDS-PAGE BOLT gels at 165V for 40 min, and transferred to a PVDF membrane (Millipore) using a semi-dry transfer system at 25 V for 25 min. The membrane was dried overnight and then blocked with 5% BSA in tris buffered saline containing 0.1% tween-20 (TBST), and proteins were detected with α-IE, α-gB, and α-GAPDH antibodies. The membrane was detected by autoradiography with chemiluminescent solution (West Pico Plus Solution, ThermoFisher, Waltham, MA, USA). For the detection of HiBiT-tagged proteins, the blots were washed for 1 min in 0.1% TBST, and placed in HiBiT detection buffer with LgBiT protein (Promega, Madison, WI, USA), at a 1:200 dilution and rocked at room temperature for 1 h. The NanoBiT substrate (1:500) was then added to the solution and the blot was rocked at room temperature for 5 min. Luminescent signal was detected by autoradiography.

HiBiT lytic detection system: Samples of viral or cellular lysates (25 µL) were combined with 25 uL of HiBiT lytic detection mix (1× buffer, 1:100 LgBiT, 1:50 substrate; Promega, Madison, WI, USA), in a black-walled 96-well plate, and rocked in the dark at room temperature, for 10 min. The luminescence of samples was determined using a Synergy HTX multi-mode microplate BioTeK plate reader, with a gain of 135.

HiBiT Co-precipitation: Equal quantities of isolated viral particle preparations, normalized to gB, were incubated for 15 min at 4 °C in 1 mL 1× cell lysis buffer (Cell Signaling, Danvers, MA, USA), without protease inhibitors to lyse the viral particles. An untreated aliquot was kept for determining the input levels of protein by Western blotting. Following incubation samples were vortexed thoroughly and spun at 9167× *g* at 4 °C for 10 min. Clarified lysates were transferred to clean the 1.5 mL tubes and HaloTag-LgBit protein (ProMega, Madison, WI, USA) was added at 1:100. Samples were incubated with occasional mixing for 1 h at 4 °C. Prior to addition, Magne HaloTag beads (ProMega, Madison, WI, USA) were washed 4 times in 0.05% NP-40 in TBS, with 1 mL/wash. The HaloTag-LgBiT viral lysate mixture was added to the HaloTag beads (40 µL/sample) and rocked overnight at 4 °C. Following incubation, supernatants were removed and kept as the unbound fraction. Beads were washed 4 times in 0.05% NP-40, in TBS, with 1 mL/wash. Excess wash buffer was removed and 40 μL of 2× NuPage loading buffer with 2% BME was added to the beads. Initial samples, as well as bound and unbound fractions were mixed 1:1 with 4× NuPage loading buffer, with 2% BME. All samples were boiled for 10 min. Samples were then loaded onto 4–12% BOLT SDS-Page gels and transferred via a semi-dry transfer system to PVDF membranes. Western blots were performed as described above for gB, R129, and HiBiT.

Viral DNA detection: Equal volumes of isolated viral particle preparations were diluted to 200 μL with PBS. DNA was extracted with the GeneJet Viral DNA and RNA purification kit (Thermo Scientific, Waltham, MA, USA) and resuspended in 50 μL of the eluent. Serial 10-fold dilutions of extracted DNA were prepared with molecular grade water; and qPCR was performed using primers and probe that target the RCMV DNA polymerase gene (R54): forward primer: CCTCACGGGCTACAACATCA; reverse primer: GAGAGTTGACGAAGAACCGACC; probe: CGGCTTCGATATCAAGTATCTCCTGCACC. qPCR was performed using the TaqMan Fast Advanced Master Mix (ThermoFisher 4444963). RCMV viral DNA at known genome concentrations served as the quantification standard. Samples were analyzed using a QuantStudio 7 Flex Real-Time PCR system. 

Northern blot analysis: RFL6 cells were plated in 6-well dishes at 5 × 10^5^ cells/well and infected in duplicate with RCMV at an MOI = 1, or left uninfected as a control. Additional duplicates were treated with foscarnet at 0.5 mM and infected with RCMV at an MOI of 1. At 8, 24, and 48 hpi, cell lysates were harvested in TRIzol. RNA was extracted from the infected cells using the Trizol method. In brief, cells were washed and then incubated with 1 mL TRIzol; and then the sample was collected. The Trizol samples were loaded onto phase-lock tubes with 200 μL of 2-bromo-3-chloropropane and mixed by inversion. Tubes were centrifuged for 5 min at 20,000× *g*. Aqueous phase was transferred to a fresh Eppendorf tube with 500 μL isopropanol and 2 μL linear acrylamide. Samples were incubated at room temperature for 10 min, and centrifuged at 4 °C for 30 min at 20,000× *g* to pellet nucleic acids. Pellets were washed twice in 75% ethanol and resuspended in molecular-biology grade water. RNA samples were treated with TURBO DNase (Invitrogen, Carlsbad, CA, USA), using the manufacturer’s protocol, and then analyzed by spectrophotometry. Equal quantities of RNA were loaded onto a 1% agarose/formaldehyde gel and electrophoresed. RNA was transferred to positively charged nylon transfer membranes (GE Healthcare, Chicago, IL, USA) and then subjected to UV-crosslinking. The membrane was pre-hybridized in DIG easy Hyb (Roche, Basel, Switzerland). The blots were hybridized with a probe specific for R131, generated using a PCR DIG probe labeling kit (Roche) in DIG easy Hyb. The blots were washed with low stringency wash (2xSSC with 0.05% SDS), followed by a high stringency wash (0.1xSSC with 0.1% SDS). Anti-DIG antibody was detected after exposure to an autoradiography film, using intensifying screens.

Detection of R131 transcripts by PCR: RNA samples were generated for the Northern blot analysis, as described above, along with cDNA. A total of 800 ng of RNA was DNase-treated using the TURBO DNase-free kit (Ambion, Austin, TX, USA) and cDNA was generated using Superscript IV (Invitrogen, Carlsbad, CA, USA). A total of 0.5 μL of cDNA was used for a PCR reaction with 25 cycles and an extension time of 1 min, with Platinum HiFi PCR master mix (ThermoFisher, Waltham, MA, USA), using 1 μL of each primer at 10 μM. R131 primers were P1: 5′-GCTTTGGGTATCGTCGAATG-3′ and P2: 5′-AGAATAGCCGTTCGGAATAG-3′. Ladder used was 1 kb plus protein ladder (ThermoFisher, Waltham, MA, USA). RCMV DNA extracted for viral DNA detection was used as a positive control and PCR-grade water was used for the no template control, as described above.

Viral entry assays: Cells were plated at 2 × 10^4^ (RFL6), 4 × 10^4^ (vSMC), 5 × 10^4^ cells/well (Dendritic cells, Bone Marrow, Macrophages), and 1 × 10^4^ cells/well (Epithelial cells), and allowed to recover overnight. Cells were infected with RCMV WT or mutants in triplicate wells at an MOI = 0.5. At 20 hpi, cells were fixed with 3.7% PFA, permeabilized with 0.15% Triton X-100, and stained with α-IE (1:250). After washing, the cells were incubated with an α-Rabbit secondary conjugated to AlexaFluor 594 (1:1000) and counterstained with DAPI. Cells were imaged using an EVOS scanning scope at 10× magnification. Total cells per field of view were counted based on the DAPI staining of nuclei, with an average number of cells per well counted, as shown in Table 4, and the RCMV positive cells were determined via α-IE staining.

## Figures and Tables

**Table 1:** Cytomegalovirus (CMV)-encoded chemokines.

Name	Known Receptors	Possible Homologues	Functions	Classification	References
UL128	R129 receptors: CCR3, CCR4, CCR5, CCR7	R129/m129	Entry, regulation of leukocyte recruitment	CC	[13,14,15]
UL130	Unknown	R131/m131	Entry, Macrophage recruitment, promotion of inflammation, viral dissemination	XC	[15,16,17]
UL146	CXCR1, CXCR2	No known homologues in RCMV or MCMV	Neutrophil recruitment, viral dissemination	CXC	[18,19,20]
UL147	Unknown	No known homologues in RCMV or MCMV	No known function	CXC	[20]
RCMV- vXCL1	XCR1		Dendritic Cell Recruitment	C	[20]
MCMV - MCK-2	CCR-2	eCK-2, RCK-2	Slow viral clearance	CC	[15,21,22]
RCK-3	Unknown		Unknown	CC	[15,16,22,23]

**Table 2:** CMV-encoded chemokine receptors.

Name	Known Ligands	Possible Homologues	Function	References
**US27**	Unknown	RhCMV -214, -215, -216, -218, -220	No known functions	[24]
**US28**	CCL2, CCL3, CCL4, CCL5, CX3CL1	RhCMV -214, -215, -216, -218, -220	Immune modulation, viral entry or cell tropism, cellular migration, signaling, viral latency and reactivation	[24,25]
**UL33**	β-chemokine receptor; m33 ligands: mCCL5	R33/M33	CREB activation, cell migration, necessary for replication *in vivo*	[15,26,27]
**UL78**	Unknown	R78, M78, homologues present in all CMVs	Viral replication	[15,28]

**Table 3:** CMV pentameric entry complex determinants.

	Putative Pentamer Components	Fibroblasts	Macrophages & Monocytes	Endothelial Cells	Epithelial Cells
**Human CMV**	gH/gL/UL128/UL130/UL131A	Not required	Required	Required	Required
**Rhesus CMV**	gH/gL/Rh157.5/Rh157.4/Rh157.6	Not required	?	Required	Required
**Guinea Pig CMV**	gH/gL/GP129/GP131/GP133	Not required	Required	Required	Impaired entry
**Mouse CMV**	gH/gL/MCK-2	Not required	Required	?	Not required
**Rat CMV**	gH/gL/R129/R131/?	Not required	Required	?	Impaired entry

**Table 4:** Average number of cells per well counted for RCMV entry assays.

	RFL6	vSMC	Epithelial Cells	Bone Marrow	BMDC	BMDM
Uninfected	806.3	550.0	395.3	151.3	586.7	77.7
WT	801.0	524.3	429.3	270.3	528.3	78.0
R131 2 × STOP	614.0	457.0	411.0	154.3	560.7	163.7
R131 C36A	804.3	12.3	434.7	269.7	747.7	88.7
R131 ΔCT	781.0	645.0	466.7	123.7	563.0	63.3
R129 2 × STOP	813.7	14.3	392.7	55.0	676.3	241.7
R129 ΔCT	735.3	468.7	394.3	153.7	671.3	87.7
2×/2× STOP	839.0	403.7	424.3	203.3	601.3	228.0

The percentage of positive cells was determined for each field of view, and all samples were normalized against WT infection for the specific cell type.
